# Room temperature continuous wave, monolithic tunable THz sources based on highly efficient mid-infrared quantum cascade lasers

**DOI:** 10.1038/srep23595

**Published:** 2016-03-24

**Authors:** Quanyong Lu, Donghai Wu, Saumya Sengupta, Steven Slivken, Manijeh Razeghi

**Affiliations:** 1 Center for Quantum Devices, Department of Electrical Engineering and Computer Science, Northwestern University, Evanston, IL 60208, USA

## Abstract

A compact, high power, room temperature continuous wave terahertz source emitting in a wide frequency range (ν ~ 1–5 THz) is of great importance to terahertz system development for applications in spectroscopy, communication, sensing, and imaging. Here, we present a strong-coupled strain-balanced quantum cascade laser design for efficient THz generation based on intracavity difference frequency generation. Room temperature continuous wave emission at 3.41 THz with a side-mode suppression ratio of 30 dB and output power up to 14 μW is achieved with a wall-plug efficiency about one order of magnitude higher than previous demonstrations. With this highly efficient design, continuous wave, single mode THz emissions with a wide frequency tuning range of 2.06–4.35 THz and an output power up to 4.2 μW are demonstrated at room temperature from two monolithic three-section sampled grating distributed feedback-distributed Bragg reflector lasers.

There has been a great deal of effort to develop compact and powerful THz sources in the 1–5 THz range for various emerging applications that ranges from biological sensing to astronomical detection^1^. Gunn diodes, resonant tunnelling diodes and Schottky diode-based frequency multipliers have demonstrated very promising compact electronic terahertz sources^2^,^3^,^4^. However, this category of THz sources has limited bandwidth, poor power efficiency and low output power level beyond 1 THz. Passive optical down-conversion to THz frequencies in bulk nonlinear materials with external pumping sources has been able to produce very high THz power at room temperature^5^. Because of the absorption in the nonlinear crystals, the effective length in which terahertz waves are generated is limited, thus their conversion efficiency is low and high-power optical pumps have been necessary to provide sufficient THz powers for applications. GaAs-based THz quantum cascade lasers (QCLs) are powerful optical semiconductor THz sources in the 2–5 THz range capable of continuous wave (CW) operation^6^,^7^. The cryogenically-cooled single mode THz QCLs can be tuned ~25 GHz at 3.3 THz by gas condensation and dielectric deposition^8^, and ~330 GHz at 3.8 THz with a THz wire waveguide coupled to a micro-electro-mechanical system^9^. Nevertheless their maximum operating temperature is still ~40 K lower than the lowest temperature reachable by a simple thermoelectric cooler^10^.

THz sources based on intracavity difference-frequency generation (DFG) from mid-infrared (mid-IR) QCLs, are currently the only semiconductor sources that are able to emit multi-mW power and cover the entire 1–5 THz range at room temperature^11^,^12^. Wide-range frequency tuning spanning several THz at room temperature based on composite distributed feedback (DFB) QCL arrays^13^, or external cavity technique^14^ has been demonstrated. Monolithic electrical tuning is more convenient for practical applications. Monolithic tuning range of 3.44 to 4.02 THz was achieved with a dual-section DFG QCL waveguide design^15^, and was further expanded to 2.6 to 4.2 THz with a three-section DFG QCL waveguide design^16^. Very recently, by utilizing a low-loss buried-ridge waveguide design and highly dissipative epi-down mounting scheme, room temperature CW operation at 3.6 THz was demonstrated with a continuous power of 3 μW^17^. However, the relatively high threshold current density and low wall-plug efficiency (WPE) of the demonstrated devices prevented the room temperature CW operation of the monolithic tunable devices because the anti-reflection coatings and lossy isolation channels are usually applied to achieve wide-range frequency tuning. The nonlinearity of THz DFG QCL devices can be further enhanced with a dual-upper-state QCL active region design^18^. The interaction between the low lasing level and the dual upper lasing levels contributes additional nonlinearity to the THz generation. Nevertheless, the rather high threshold current density of the device prevented its CW operation.

## Results and Discussion

Here we design and demonstrate a broadband, strong-coupled, strain-balanced QCL active region design with a large nonlinear susceptibility for high power CW THz emission in a wide frequency range based on DFG. Room temperature, continuous, single mode emission at 3.41 THz with a side-mode suppression ratio (SMSR) of 30 dB and output power of 14 μW is achieved, and continuous wave electrical tuning of 2.06–4.35 THz with a THz power up to 4.2 μW is demonstrated from two monolithic three-section sampled grating distributed feedback-distributed Bragg reflector (SGDFB-DBR) lasers.

Currently, all the demonstrations of THz sources based on mid-IR QCLs have been made with lattice-matched Ga_0.47_In_0.53_As/Al_0.48_In_0.52_As nonlinear active region designs in the wavelength range of λ ~ 9–11 μm^13^,^14^,^17^,^18^,^19^. The limited conduction band offset (~0.5 eV) for the lattice-matched design results in an energy spacing of ~240 meV between the upper laser level and the continuum states located above the barriers, which is not able to confine the electrons in the upper laser level effectively, and induces significant thermally activated carrier leakage into the continuum. This leads to less efficient performance of the devices compared to the state-of-art shorter-wavelength counterparts^20^,^21^.

**Figure 1:**
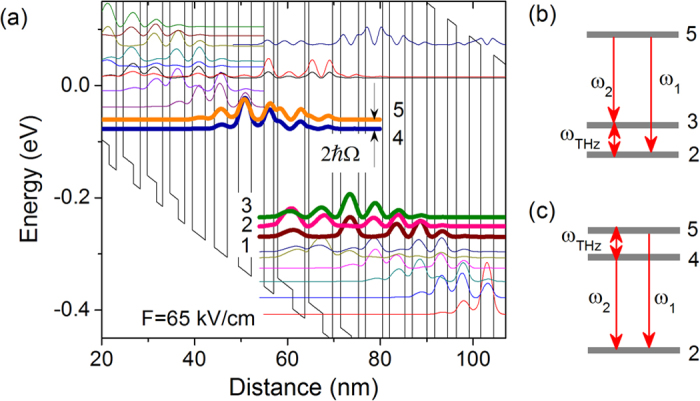
(**a**) Strain-balanced active region band structure. The design is based on Al_0.63_In_0.37_As/Ga_0.35_In_0.65_As/Ga_0.47_In_0.53_As material system. The layer sequence in nm, starting from the injection barrier, is **2.7**/ 2.1/ **0.9**/ *3.2*/ 2.6/ **0.9**/ *3.0*/ 2.1/ **1.7**/ *2.4*/ 1.5/ **1.5**/ *2.2*/ 1.5/ **1.5**/ *2.0*/ 1.3/ **1.6**/ *1.8*/ 1.3/ **1.7**/ 2.8/ **1.9**/ 2.8/ **2.4**/ 2.8. The barriers are in bold font, and the wells are in normal font, the Ga_0.47_In_0.53_As insertions are in italic, and the underlined layers are doped to *n* = 1.7 × 10^17 ^cm^−3^. (**b,c**) Schematic descriptions of two major DFG processes for the strain-balanced design, involving one upper level 5 (4) and two lower levels *j, k* (*j, k* = 1, 2, 3. *j *≠ *k*) (**b**), and two upper levels 4, 5, and one lower level *j* (*j* = 1, 2, 3) (**c**).

In the present work, a nonlinear active region based on the strain-balanced Al_0.63_In_0.37_As/Ga_0.35_In_0.65_As/Ga_0.47_In_0.53_As material system is designed with a single-phonon resonance depopulation scheme. The band structure is shown in Fig. 1(a). The targeted wavelength is λ ~ 7.8 μm. The inserted Ga_0.47_In_0.53_As layers are used to balance the material strain within one stage. The conduction band offset is enhanced to ~0.74 eV. In the present strain-balanced design with a diagonal optical transition scheme, the increased conduction-band offset and interface roughness increases the broadening the oscillation linewidth to ~15–20 meV, compared to that of ~10 meV for the lattice-matched active region design^22^. This allows for a stronger coupling design between the injector and upper lasing level. A high coupling strength with an energy splitting of 2 ħΩ = 16.5 meV is calculated for the present structure. This strong-coupling design not only effectively improves the carrier tunnelling rate into the upper lasing level 5^23^, but also enhances the DFG nonlinear susceptibility χ^(2)^. Normally, the optical nonlinearity is engineered by the adjusting the injector barrier thickness so that the lower laser level 2 couples with its neighbouring levels 1, and 3 with energy spacing ranging 14–17 meV (3.4–4.1 THz), as the schematic shown in Fig. 1(b). Here, the strong-coupling design with an energy splitting of 16.5 meV, provides another scheme to the total nonlinear susceptibility, as depicted in Fig. 1(c). Given a threshold gain *g*_*th*_ = 5 cm^−1^, a total nonlinear susceptibility of |χ^(2)^| = 2.0 × 10^4^ is obtained from the present design. (See supplementary part 1) This is comparable to the value of 2.6 × 10^4 ^pm/V from the previous lattice-matched active region design at λ ~ 9 μm with a higher threshold gain^17^.

**Figure 2:**
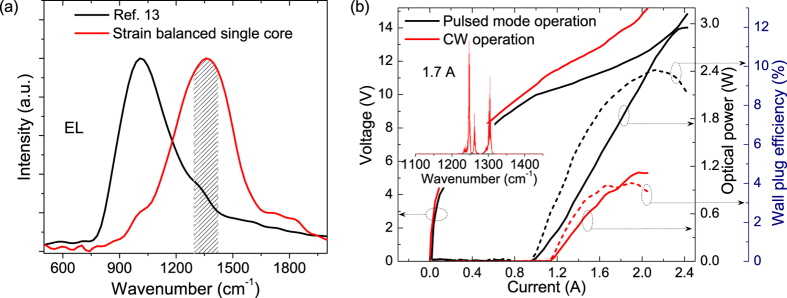
(**a**) EL spectra for the strain-balanced single-core design and the lattice matched dual-core design from ref. 13. The shaded area indicates the supported dual wavelength emissions with the frequency spacings up to 5 THz. (**b**) *P-I-V* and wall plug efficiency characterizations of a 4.9-mm long uncoated FP device in pulsed mode and CW operations at 293 K. Inset: lasing spectrum at 1.7 A in CW operation.

The other benefit of this strong-coupled strain-balanced design is the increased gain spectral width compared to the lattice matched design. The electroluminescence (EL) spectrum measured from a mesa containing 40-stage single-core strain-balanced structure grown on an n-InP substrate exhibits a full-width at half maximum (FWHM) of 380 cm^−1^ (shown in Fig. 2(a)). The EL spectrum is even broader than the previous dual-core lattice-matched active region with a FWHM of ~330 cm^−1 ^in the 9–11 μm wavelength range. The increased FWHM of EL spectra is attributed to the strong-coupled diagonal optical transition design involving multiple transitions and the increased oscillation linewidths. This single-core strain-balanced design is thus able to support 1–5 THz composite DFB designs with the designed wavelengths within 90% of the EL peak, as indicated by shaded area in Fig. 2(a).

In the experiment, a laser structure consisting of 40 stages of strain-balanced structure was grown on a semi-insulating InP substrate. Details of the growth of this control wafer are given in the methods section. Part of this wafer was processed into buried-composite-DFB, buried-ridge waveguides with double-side current extraction schemes following the procedure described in refs. 17 and 24, while the other part of the wafer without grating patterns was processed into Fabry-Pérot (FP) devices for comparison. Grating periods of *Λ*_1_ = 1.18 μm and *Λ*_2_ = 1.30 μm were used for the two components of the composite-DFB grating design. The DFB and FP devices were epi-down mounted on diamond submounts predefined with corresponding Indium patterns for efficient heat extraction^24^. The front facet of the composite DFB device was polished into 30° to collect the THz light satisfying the Čerenkov phase matching condition^25^.

Figure 2(b) is the optical power-current-voltage (*P-I-V*) characterizations of a 12-μm-wide, 4.9-mm long, uncoated FP device on a semi-insulating substrate in pulsed mode (pulse width = 200 ns and duty cycle = 2%) and CW operations at 293 K. The inset is the CW lasing spectrum at 1.7 A. The FP device exhibits a maximum power of 3 W and threshold current density of 1.95 kA/cm^2^ in pulsed mode operation, and a maximum power of 1.1 W and a threshold current density of 2.2 kA/cm^2^ in CW operation. The maximum wall plug efficiencies (WPEs) are 10% and 4% for pulsed mode and CW operations, respectively. This is compared with previous results based on latticed matched active region with maximum WPEs of 2.8% and 1% in pulsed mode and CW operations, respectively^17^. In addition, a 4.9-mm long uncoated FP device on n-substrate exhibits a pulsed power up to 5.1 W with a threshold current density of 2.19 kA/cm^−1^, and a CW power up to 1.6 W with a threshold current density of 2.5 kA/cm^−1^, respectively. The maximum WPE is 12.6% and 5% (See supplementary Fig. 1S). The relatively lower power and efficiency of the device on semi-insulating substrate is mainly attributed to the extra voltage drop due to side current injections through the bottom contact.

**Figure 3:**
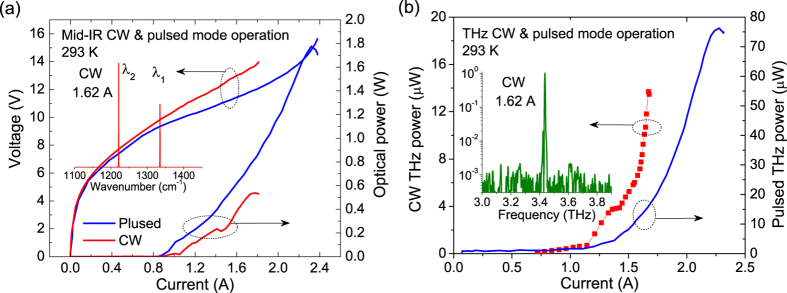
(**a**) *P-I-V* characterizations of a 4-mm long buried-ridge composite DFB device in pulsed mode and CW operations at 293 K. Inset: CW dual-wavelength operation at λ_1_ = 7.46 μm and λ_2_ = 8.15 μm with a frequency spacing of 3.4 THz at a current of 1.62 A. (**b**) THz pulsed and CW powers as functions of current. Inset: CW emitting spectrum at 3.41 THz at a current of 1.62 A.

In pulsed mode and CW operations, a 4-mm long composite DFB device emits up to 1.74 W and 0.53 W with threshold current densities of 1.70 and 1.87 kA/cm^2^, respectively, as shown in Fig. 3(a). The inset is the CW lasing spectrum at 1.62 A. Stable dual-wavelength operation at λ_1_ = 7.46 μm and λ_2_ = 8.15 μm with a frequency spacing of 3.4 THz is observed. Figure 3(b) is the THz power in pulsed mode and CW operations at 293 K. Maximum pulsed and CW powers of 76 μW and 14 μW with conversion efficiencies of η = 0.11 and 0.35 mW/W^2^, and THz WPEs of 2.6 × 10^−6^ and 0.8 × 10^−6^ are obtained, respectively. The superlinear increase in CW THz power in Fig. 3(b) is due to the rapid power increase in λ_1_ and dramatic power balancing between λ_1_ and λ_2_ in the current range of 1.45–1.68 A. The maximum CW powers are much higher than previous demonstrations in ref. 17 despite that the conversion efficiency is lower due to the relatively lower nonlinearity induced by the reduced threshold gain. As a result, the THz pulsed and CW WPEs are about 3 and 8.5 times higher than previous demonstrations. The increased THz power and WPE are mainly attributed to the enhanced mid-IR power and efficiency of the strong-coupled strain-balanced active region design.

The THz spectra are tested with a Bruker Fourier transform infrared (FTIR) spectrometer equipped with an uncooled far-IR deuterated L-alanine doped triglycine sulfate (DTGS) detector. The side mode suppression ratio (SMSR) is about 30 dB at a CW current of 1.62 A as shown the inset of Fig. 3(b). The linewidth is ~4.9 GHz, which is mainly limited by the resolution (~3.75 GHz) of the FTIR.

**Figure 4:**
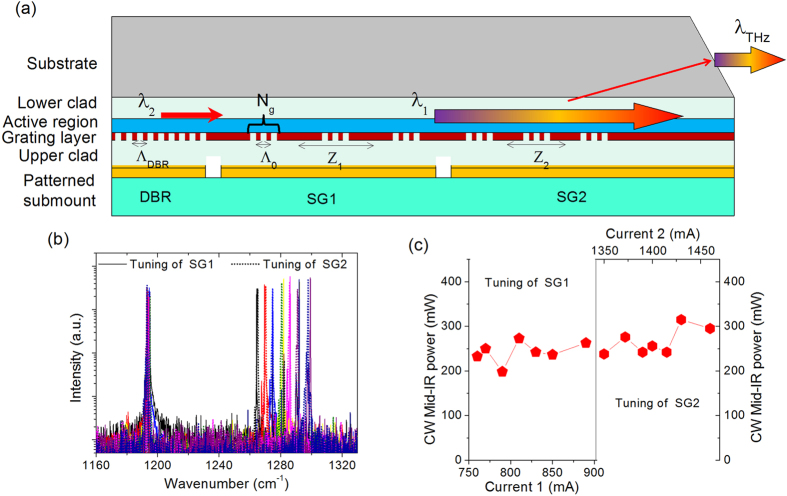
(**a**) Schematic of an epi-down mounted three-section SGDFB-DBR device. (**b**) Characterizations of the tunable mid-IR spectra of a SGDFB-DBR device by changing the DC currents on SG1 (solid lines) and SG2 (dashed lines). (**c**) The corresponding CW power in the tuning range by changing the DC currents on SG1 with a fixed current of 1390 mA on SG2 (left part of (**c**)), and changing the DC currents on SG2 with a fixed current of 830 mA on SG1 (right part of (**c**)), respectively.

To verify the broadband gain and nonlinearity design of this structure, another sample from the same wafer is processed into a three-section SGDFB-DBR waveguide, as shown in Fig. 4(a). Both the two sampled grating (SG) sections are sampled with a very short grating section (*Λ*_*0*_ = 1.22 μm, *N*_*g*_ = 15) for 7 times. Here *N*_*g*_ is the grating number in one short section. The sampling periods *Z*_1_ = 207 μm and *Z*_2_ = 185 μm were used for the front and back SG sections, respectively. To enhance the power performance, the front SG section was further elongated with a 1.5-mm unpatterned section for power amplification. Laser bars with 6.3-mm cavity length were cleaved, containing one 1-mm DBR section (*Λ*_*DBR*_ = 1.329 μm) on the back, one 2-mm SG section (SG1) in the middle, and one 3.3-mm SG section plus the amplifier (SG2) in the front. These three-section devices are then epi-down mounted on patterned diamond submounts for CW measurements.

All three sections are biased with independent DC drivers for electrical tuning. Sections SG1 and SG2 were biased to DC currents of 0.83 A (1.5 *J*_*th*_*S*_1_) and 1.39 A (1.52 *J*_*th*_*S*_2_), respectively, and tuning was accomplished with additional changing currents applied to one or both sections. Here *J*_*th*_  = 2.31 kA/cm^2^ refers to the threshold current density of the three sections pumped simultaneously by one DC driver. *S*_m_ (*m* = 1, 2, 3) are the device areas for sections SG1, SG2, and DBR. The DBR section was independently biased at a constant low DC current, i.e., 150–250 mA (~0.67–0.95 *J*_*th*_*S*_3_) to lower the absorption loss, yet still below the threshold current to prevent the self lasing. The wide tuning for the shorter wavelength *λ*_1_ was realized mainly through Vernier tuning. In other words, by changing the DC currents on the two SG sections, the lasing spectrum was rapidly tuned among the different supermodes. The tuning of *λ*_1_ is achieved by varying the DC currents of SG1 section with a current step of 10–25 mA, as shown in Fig. 4(b). Wavelength tuning from 7.7 to 7.91 μm (~35 cm^−1^) with SMSRs of 30–35 dB in the tuning range is realized. The Vernier tuning step size is 5.8–6.1 cm^−1^. A similar tuning range with a smaller tuning step of 5.3–5.6 cm^−1^ was observed by changing the DC current on SG2 section by a current step of 15–35 mA, as plotted in dashed lines in Fig. 4(b). The corresponding CW mid-IR powers in the tuning ranges by tuning the two sections, are ~200–315 mW, as shown in Fig. 4(c).

**Figure 5:**
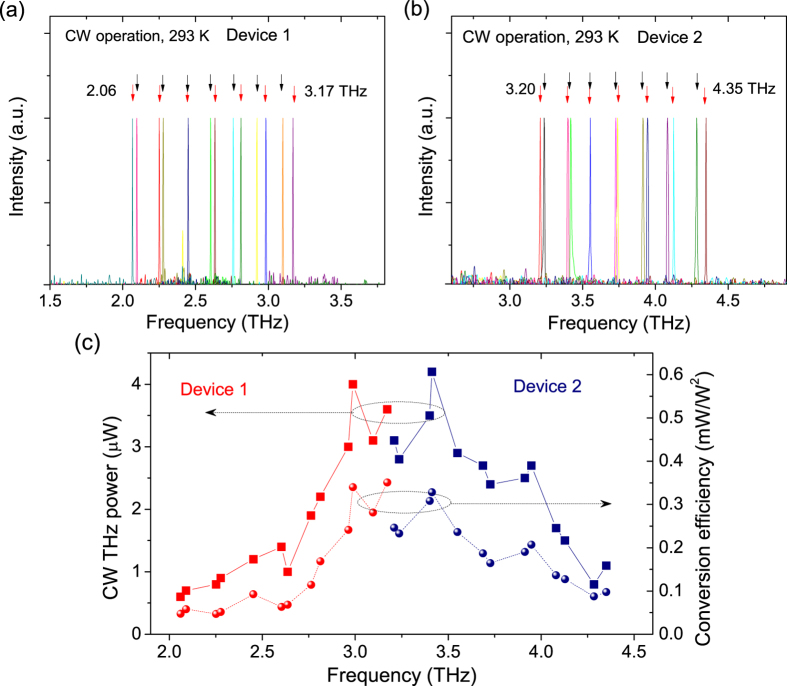
(**a,b**) Characterizations of THz spectral tuning for the two SGDFB-DBR devices designed with different frequency spacings. The black arrows indicate the tuning by changing currents on SG2, and the red arrows indicate the frequency tuning by changing currents on SG1. (**c**) The corresponding CW THz power and conversion efficiency in the tunable THz frequency range for the two monolithic devices.

The measured THz tuning spectra are presented in Fig. 5(a). A wide frequency tuning from 2.06 to 3.17 THz with a step of 185 GHz by changing the DC current on SG1, and 2.09 to 3.1 THz with a step of 168 GHz by changing the DC current on SG2, are achieved respectively. Another device with a slightly different grating period designs of *Λ*_*0*_ = 1.196 μm and *Λ*_*DBR*_ = 1.36 μm targeting a larger frequency spacing, exhibits a full tuning range of 3.2 to 4.35 THz, as shown in in Fig. 5(b). The combined tuning ranges for both devices is 2.29 THz. The SMSR ranges from 12 to 25 dB in the tuning range measured with an uncooled far-infrared DTGS detector. When using Liquid-Helium cooled Silicon bolometer, the SMSRs of these tunable THz sources should be only limited by the mid-IR SMSRs (30–35 dB) owing to the reduced background noise in the Silicon bolometer and a much higher sensitivity than the DTGS detector. The spectral linewidths are ~4–5 GHz in the tuning range, which are much narrower than the linewidth of ~10 GHz from the pulsed mode operating THz sources^14^,^15^. Considering a linewidth of 150 kHz from a free-running CW mid-IR QCL^26^, the THz spectral linewidth of our CW devices should have similar linewidths on the order of hundreds of kHz, which makes the devices ideal for the applications in high-resolution THz spectroscopy^27^.

In the tuned THz frequencies, the THz output power ranges from 0.6 μW at 2.06 THz to 4.2 μW at 3.42 THz, as shown in Fig. 5(c). The THz power performance as a function of frequency is related mainly to the conversion efficiency characteristic of the devices, as shown in Fig. 5(c). The conversion efficiency peaks around 3.0–3.5 THz with η_max_ = 0.36 mW/W^2^ at 3.2 THz, and decreases towards the lower frequency end due to the decreasing nonlinearity and decreases towards the higher frequency end due to the increasing optical loss in the InP substrate^13^. Note the present tuning was achieved at a relatively low current density of 1.4–1.5*J*_*th*_ compared to the roll-over current density of 2.1*J*_*th*_. A simple way to increase the power is to use a higher current baseline for the tuning, which will increase the power quadratically. Also the present three-section device has two 100-μm isolation channels. The optical absorption in these regions greatly reduces the output power. When using a waveguide design with narrower isolation channels (e.g., 5–10 μm wide)^28^, the output mid-IR power can be improved significantly. On the other hand, since the conversion efficiency experiences a less variation as the function of temperature^29^, high CW THz power up to ~0.1 mW is expected when cooling the device to a temperature of 250 K which is attainable by a TEC cooler. Nevertheless, higher THz power in a wider tuning range in CW operation at room temperature is achievable by further exploring the active nonlinear region design in the 5–6 μm wavelength range with a larger coupling strength and higher WPE^30^,^31^, and a digital concatenated sampled grating for a wider tuning range per device^32^.

## Conclusions

In conclusion, we report a room temperature CW THz source based on a strain-balanced mid-IR quantum cascade laser at 3.41 THz with a SMSR of 30 dB and output power up to 14 μW. The CW THz wall plug efficiency is enhanced by one order of magnitude compared with the previous demonstrations. With the improved active structure, the same laser wafer is used to demonstrate room temperature, CW single mode THz emissions with a wide tunable frequency range of 2.06–4.35 THz and THz power up to 4.2 μW from two monolithic three-section SGDFB-DBR lasers. The demonstrated CW tunable compact THz sources with narrow linewidths and improved efficiency will make them possible for real-world applications, such as spectroscopy and sensing.

## Methods

### Growth and fabrication

The QCL structure presented in this work is based on the strain-balanced Al_0.63_In_0.37_As/Ga_0.35_In_0.65_As/Ga_0.47_In_0.53_As material system grown by gas-source molecular beam epitaxy (MBE) on a semi-insulating InP substrate. The growth started with a 200-nm InGaAs layer (Si, ~1 × 10^18 ^cm^−3^), and 3-μm InP buffer layer (Si, ~2 × 10^16 ^cm^−3^). The laser core consisted of a 40 stage strain-balanced single-phonon resonance (SPR) structure. The design features a broad gain spectrum and a giant nonlinear susceptibility for THz DFG. The average doping of the active region is ~2.1 × 10^16 ^cm^−3^. The growth ended with a 400-nm-thick InGaAs grating layer (Si, ~2 × 10^16 ^cm^−3^) and a 10-nm-thick InP cladding layer (Si, ~2 × 10^16 ^cm^−3^). A control wafer with identical active structure is grown on an n-InP substrate with a slightly higher average active region doping of ~2.5 × 10^16 ^cm^−3^.

Two pieces from the semi-insulating wafer are defined with composite DFB and SGDFB-DBR gratings by e-beam lithography and dry etching for high THz power and monolithic tuning, respectively. After the grating fabrication, The wafers were regrown with 4.5-μm InP cladding (Si, 2–5 × 10^16 ^cm^−3^) and 0.5-μm InP (Si, 5 × 10^18 ^cm^−3^) cap layers on the grating layer by metal organic chemical vapor phase deposition (MOCVD). The samples are then processed into planarized buried heterostructure ridge waveguide with ridge width of 12–13 μm. Bottom contacts for side current injection are defined on the sides of the laser ridge by selective wet etching. The top contact area is formed via a window etched into the 500-nm Si_3_N_4_ insulating layer on top of the laser ridge. Ti/Au (40 nm/120 nm) is used for both top and bottom contacts. An additional 4 μm of Au is electroplated on top of the QCL wafer for better heat spreading. For monolithic tuning, two 100-μm wide isolation channels are defined between the three sections of SGDFB-DBR design for independent electrical tuning. The backside of the substrate was left unprocessed since the substrate is insulating and an epilayer-down mounting scheme is to be used for testing. The sample with composite DFB gratings is cleaved into 4 mm long laser cavities and the back facet is high-reflection (HR) coated with Si_3_N_4_/Au (400/100 nm). SGDFB-DBR devices are cleaved into 6.3 mm long devices and anti-reflection (AR) coated with 800-nm Y_2_O_3_ on the front facet. Both devices are epilayer-down mounted on patterned diamond submounts, and the front facets are polished at a 30° angle with respect to the cleaving plane for THz outcoupling. As comparison, the control wafer is processed into a planarized buried heterostructure ridge waveguide with a 12-μm ridge width. A laser bar with 4.9-mm cavity length is cleaved and epilayer-down mounted on a diamond submount for testing.

### Device Testing

All measurements were performed at room temperature. Output mid-IR power in pulsed mode and CW operations was measured using a calibrated thermopile detector for the average power and the peak power was obtained from the measured average power and the known duty cycle, assuming 100% collection efficiency. Golay cell detector (Microtech Instruments) was used for THz power measurement. Filters were used to eliminate the mid-IR radiation. In order to be compatible with Golay cell detection, the device was first biased up to a DC current close to the threshold, e.g., 0.7 A. Then a low frequency pulse (quasi-CW, 40 ms pulse width and 12.5 Hz repetition rate) was applied to modulate the THz signal. The power value was not corrected for collection efficiency. Spectral measurements were performed with a Bruker Fourier transform infrared (FTIR) spectrometer equipped with an uncooled mid-IR DTGS and far-infrared DTGS detectors for mid-IR and THz measurements, respectively. Spectra were taken in rapid scan mode at a resolution of 0.125 cm^−1^ (3.75 GHz).

## Additional Information

**How to cite this article**: Lu, Q. *et al*. Room temperature continuous wave, monolithic tunable THz sources based on highly efficient mid-infrared quantum cascade lasers. *Sci. Rep.* **6**, 23595; doi: 10.1038/srep23595 (2016).

## Supplementary Material

Supplementary Information
