# Supplementary material for: Room temperature continuous wave, monolithic tunable THz sources based on highly efficient mid-infrared quantum cascade lasers
Source: Sci Rep. 2016 Mar 24;6:23595. doi: 10.1038/srep23595 (PMC4806356; doi:10.1038/srep23595)
Supplement: Supplementary Information [file srep23595-s1.pdf]

# Room temperature continuous wave, monolithic tunable THz sources based on highly efficient mid-infrared quantum cascade lasers

Quanyong Lu, Donghai Wu, Saumya Sengupta, Steven Slivken, & Manijeh Razeghi*

*Center for Quantum Devices, Department of Electrical Engineering and Computer Science, Northwestern University, Evanston, IL 60208, USA.* *razeghi@eecs.northwestern.edu

## Supplementary Material

1. Calculation of the nonlinear susceptibility $\chi^{(2)}$ for strain-balanced active region design

Coupling strength ħΩ between the injector level 4 and upper lasing level 5 governs the resonant current density $J$, as seen from the following equation[1, 23]:

$$J = eN_s \frac{2\Omega^2 \tau_\perp}{1+\Delta^2\tau_\perp^2 + 4\Omega^2 \tau_{ul}\tau_\perp} \quad (1)$$

where $N_s$ is the sheet doping density, ħΔ is the energy detuning of tunnelling transition, $\tau_{ul}$ is the upper level transition time to the lower level, $\tau_\perp$ is the momentum relaxation time in the quantum well plane. The coupling should be strong enough for efficient carrier injection to the upper level and less susceptibility to the energy detuning to the resonance tunnelling condition, yet it should not exceed the transition broadening to avoid the splitting of the gain.

In the present strain-balanced material system with a diagonal-transition active region design, the increased conduction-band offset and interface roughness increases the broadening of the transition linewidth to 15-20 meV, compared to that of ~10 meV for the lattice-matched active region design. This allows for a stronger coupling design between the injector and upper lasing level. A high coupling strength with an energy splitting of $2\hbar\Omega$ =16.5 meV is used in current design.

The strong-coupled strain-balanced design brings two type schemes contributing to the DFG nonlinear susceptibility $\chi^{(2)}$ which involves the strong coupling between lower laser levels 1, 2, and 3 (Fig. 1(b)), and the strong coupling between the upper laser levels 4 and 5 (Fig. 1(c)). Therefore, $\chi^{(2)}$ is rewritten as[29]:

$$
\begin{aligned}
&\chi^{(2)}\left(\omega_{THz}=\omega_1-\omega_2\right)\approx\\
&\frac{e^3}{\hbar^2\varepsilon_0}\sum_{j,k=1,2,3;\,j\neq k}^{m=4,5}\frac{z_{mj}z_{jk}z_{km}}{\omega_{THz}-\omega_{jk}+i\Gamma_{jk}}\left[\frac{N_m-N_j}{\omega_1-\omega_{mj}+i\Gamma_{mj}}+\frac{N_m-N_k}{-\omega_2+\omega_{mk}+i\Gamma_{mk}}\right] \qquad (2)\\
&+\frac{e^3}{\hbar^2\varepsilon_0}\sum_{j=1,2,3}\frac{z_{j5}z_{54}z_{4j}}{\omega_{THz}-\omega_{54}+i\Gamma_{54}}\left[\frac{N_5-N_j}{\omega_1-\omega_{5j}+i\Gamma_{5j}}+\frac{N_4-N_j}{-\omega_2+\omega_{4j}+i\Gamma_{4j}}\right]
\end{aligned}
$$

Here $j$, $k$=1, 2, 3 and $m$=4, 5 represent the energy levels contributing the DFG nonlinearity. $N_m$ is the population density distribution on level $m$. $z_{mj}$, $\omega_{mj}$, and $\Gamma_{mj}$ are the dipole matrix element, frequency, and broadening of the transition between levels $m$ and $j$. $\omega_1$, $\omega_2$ , and $\omega_{THz}$ are the wave-vectors for the emitting wavelengths $\lambda_1$, $\lambda_2$, and $\lambda_{THz}$, respectively.

The increased transition broadening $\Gamma_{mj}$ in the strong-coupled strain-balanced active region design will not affect $\chi^{(2)}$ significantly compared with the lattice matched

design[17]. This is because the increased $\Gamma_{mj}$ also requires an increased population inversion $\Delta N$ to satisfy the same threshold gain while $\chi^{(2)}$ is in proportional to $\Delta N$. For our single core active region design, the gain is written as:

$$g(\omega)=\sum_{j,m}^{j\neq m}\frac{e^2\omega_{jm}^2}{c\varepsilon_0 n_{eff}\omega}\frac{z_{jm}^2\Gamma_{jm}}{\left(\hbar\omega_{mj}-\hbar\omega\right)^2+\Gamma_{mj}^2}\left(N_m-N_j\right) \tag{3}$$

Here $n_{eff}$ is the effective refractive index. The total gain $g(\omega)$ is obtained by sum all the possible transitions between different levels $j$ and $m$ ($j$, $m$=1, 2, ... $j\neq m$). Because the two wavelengths ($\lambda_1$ and $\lambda_2$) are defined on the two sides of the gain peak at $\lambda_{peak}$, the threshold condition for $\lambda_1$ which is shorter than $\lambda_2$ and has a lower free carrier absorption, can be expressed as:

$$g_{th}\left(\omega_1\right)=Tg_{th}\left(\omega_{peak}\right)=\frac{\alpha_w+\alpha_m}{\Gamma} \tag{4}$$

Here $T$ is the ratio of the gain at $\lambda_1$ respect to the maximum gain at $\lambda_{peak}$. $T\approx$0.9-0.95 is estimated for the frequency spacing of 3-4 THz between $\lambda_1$ and $\lambda_2$. $\Gamma$ is the modal confinement factor. Given a waveguide loss $\alpha_w$= 1.2 cm$^{-1}$ and mirror loss $\alpha_m$= 1.8 cm$^{-1}$ for a 4-mm long cavity with high-reflection (HR) coating, $\Gamma_{mj}$ =15 meV for the mid-IR and THz transitions, modal confinement factor $\Gamma$= 60%, and also assuming all the lower laser levels 1-3 are depleted, $\Delta N=8\times10^{14}$ and $6.5\times10^{14}$ cm$^{-3}$ were estimated. Therefore, $\chi^{(2)}$= (1.22-0.62i)$\times10^4$ and (-0.2-1.04i)$\times10^4$ pm/V are calculated for the two schemes (depicted in Fig. 1(b) and (c)) contributing to the nonlinearity. Thus a total $|\chi^{(2)}|$= 2.0$\times10^4$ pm/V is obtained from the present design. Here, a transition broadening $\Gamma_{jk}$=5 meV for THz transitions is used in the calculation.

2. Mid-IR performance of a buried-ridge FP device based on n- substrate

A 4.9-mm long uncoated FP device on n-substrate is epilayer-down mounted and tested as a reference. The device exhibits a pulsed power up to 5.1 W with a threshold current density of 2.19 kA/cm$^{-1}$, and a CW power up to 1.6 W with a threshold current density of 2.5 kA/cm$^{-1}$, respectively, as shown in Figure 1S. The maximum WPE is 12.6% and 5%, respectively.

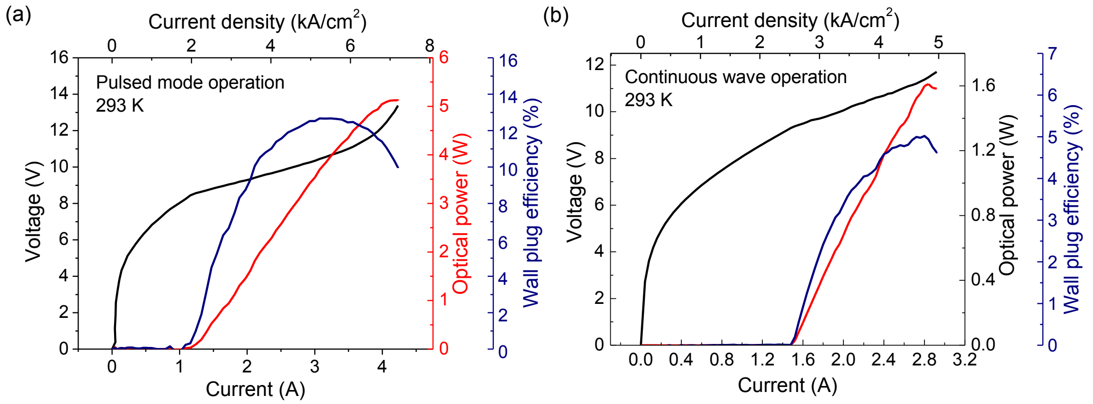


**Fig. S1. *P-I-V* and wall plug efficiency characterizations of a 4.9-mm long uncoated FP device based on n- substrate in pulsed mode (a) and (b) CW operation at 293 K.**

Reference:


1. Sirtori, C. et al. Resonant tunneling in quantum cascade lasers. IEEE J. Quantum Electron. 34, 1722–1729 (1998).
